# Empowering mothers

**DOI:** 10.3402/gha.v9.34406

**Published:** 2016-12-19

**Authors:** Tanya Seshadri, Prashanth Nuggehalli

**Affiliations:** 1 Vivekananda Gorukana Kalyana Kendra (VGKK), Chamarajanagar, India; 2 Faculty, Institute of Public Health, Bangalore, India

**Figure:**
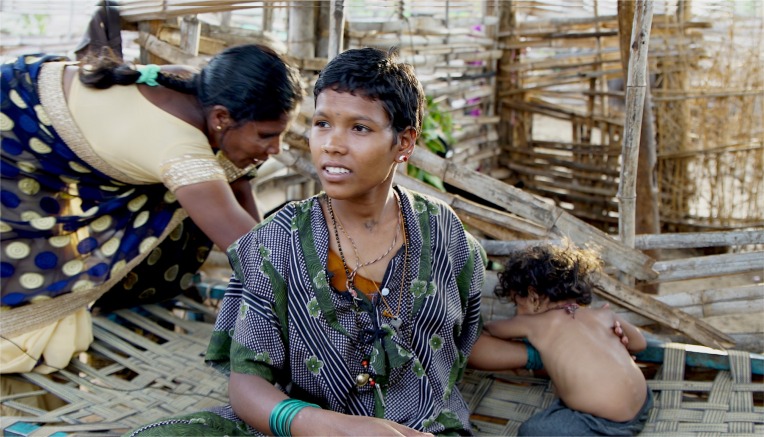
Photograph: Munmun Dhalaria, documentary filmmaker.

The image was taken on May 15, 2016 in Nagaratna's house near Male Mahadeshwara (MM) Hills wildlife sanctuary. It stands out as one of the constant reminders for the importance of empowering marginalized women into theorizing their own problems instead of relying on knowledge that is unquestioningly thrust upon them. Transforming them into the researcher is not only ethical but also crucial for public policy implementation.

Madevi is a community coordinator and a woman tribal leader from the Soliga community living in Biligirirangana Hills, Karnataka, India. She is featured here helping Nagaratna, who is being interviewed by me while trying to keep her baby calm. Nagaratna also belongs to the same indigenous community (Soliga community) as Madevi and, due to her presence, shares the story of her complicated childbirth with us. Nagaratna was pregnant at the age of 18. She travelled from her village in MM Hills to the local public health centre in a tempo. After 2 days of severe back pain, she was shifted to the taluk hospital at 3 a.m. in a jeep. Due to the ingestion of amniotic fluid by her baby, she was moved to Mysore where the baby finally got treated. She was sent home the same day in a bus. Her family now owes $200 to the families that loaned it to them during the emergency.

This happens to be a positive story as Nagaratna has already overcome the many social determinants that could hinder her chances to safe maternal health care. Living on the periphery of a wildlife sanctuary and earning less than $2 a day, she at least is not dealing with severe anaemia or an inability to access some form of public transportation to a far-off hospital, however uncomfortable.

Many more such stories of mostly negative outcomes were documented for a film that I worked on for a research being carried out by The Malki Initiative titled ‘Participation for local action implementation research with indigenous communities in southern India for local action on improving maternal health services’. It is supported by WHO Alliance for Health Policy and Systems Research.

This research brought together non-governmental organizations, community representatives, the government district health team and public health researchers and has empowered the community through an equitable partnership.

*Tanya Seshadri* Vivekananda Gorukana Kalyana Kendra (VGKK) Chamarajanagar, India Email: tanya@malki.in*Prashanth Nuggehalli* Faculty, Institute of Public Health Bangalore, India

